# Fourier Transform Infrared (FTIR) Spectroscopy as a Tool to Characterize Exercise and Physical Activity: A Systematic Review

**DOI:** 10.1007/s40279-024-02139-5

**Published:** 2024-11-20

**Authors:** Pedro Afonso Valente, Sandra I. Mota, Ana Teixeira, Elisabete Ferreiro, Hugo Sarmento, Inês Cipriano, João R. Campos, Luís Rama, Paulo J. Oliveira

**Affiliations:** 1 CNC-Center for Neuroscience and Cell Biology, University of Coimbra, Coimbra, Portugal; 2 CIBB - Center for Innovative Biomedicine and Biotechnology, University of Coimbra, Coimbra, Portugal; 3 University of Coimbra, Research Unit for Sport and Physical Activity, Faculty of Sport Sciences and Physical Education, Coimbra, Portugal; 4 University of Coimbra, Centre for Informatics and Systems of the University of Coimbra, Department of Informatics Engineering, Coimbra, Portugal

## Abstract

**Background:**

Over the past few decades, the scientific community has recognized the impact of physical activity on health and performance. In parallel, researchers have been actively exploring novel methodologies to analyze the physiological and metabolic responses to exercise. Fourier transform infrared spectroscopy has emerged as a powerful tool in this effort, offering the potential to provide unique insights into exercise-related changes at the molecular level.

**Objective:**

The primary goal of this systematic review is to confirm the viability of utilizing Fourier transform infrared spectroscopy for the analysis of the biochemical changes associated with physical exercise and its potential applications.

**Methods:**

This systematic review adhered to PRISMA (Preferred Reporting Items for Systematic Reviews and Meta-Analyses) guidelines and examined studies employing Fourier transform infrared spectroscopy to analyze exercise and physical activity, focusing on a biological sample collection and spectral analysis. Four databases (PubMed, SPORTDiscus, Web of Science, and Scopus) were searched, and inclusion criteria encompassed original English-language studies involving human participants aged 18–50 years, a biological sample collection (urine, saliva, and blood), and the use of Fourier transform infrared spectroscopy. The studies were analyzed considering the type of exercise or sport that was investigated, and also the type of spectral analysis conducted.

**Results:**

The review encompassed 15 studies that demonstrated the versatility of Fourier transform infrared spectroscopy in assessing various aspects of exercise, including metabolism, cardiovascular responses, and muscular fatigue. The largest study evaluated 57 athletes from several different sports. On average, almost all the studies were performed with around 20 athletes. Notably, the technique’s holistic approach allows for a comprehensive analysis of the complex network of metabolites and proteins within the human body. Data analysis methodologies, particularly when coupled with machine learning, show great potential for advancing the field of sports science.

**Conclusions:**

Fourier transform infrared spectroscopy emerges as a promising tool for monitoring and enhancing the performance of high-level athletes, preventing overtraining or even over-reaching, and assessing metabolism. Its accuracy, efficiency, and affordability also make it a candidate for broader applications in assessing the health and fitness of the general population. Future research should explore its applicability across diverse exercise modalities and demographic groups, aiming to prescribe exercise plans that consider a multitude of parameters for larger, more intricate exercise cohorts.

**Clinical Trial Registration:**

The study protocol was registered in the International Prospective Register of Systematic Reviews (PROSPERO) under the ID number CRD42023441965.

**Graphical Abstract:**

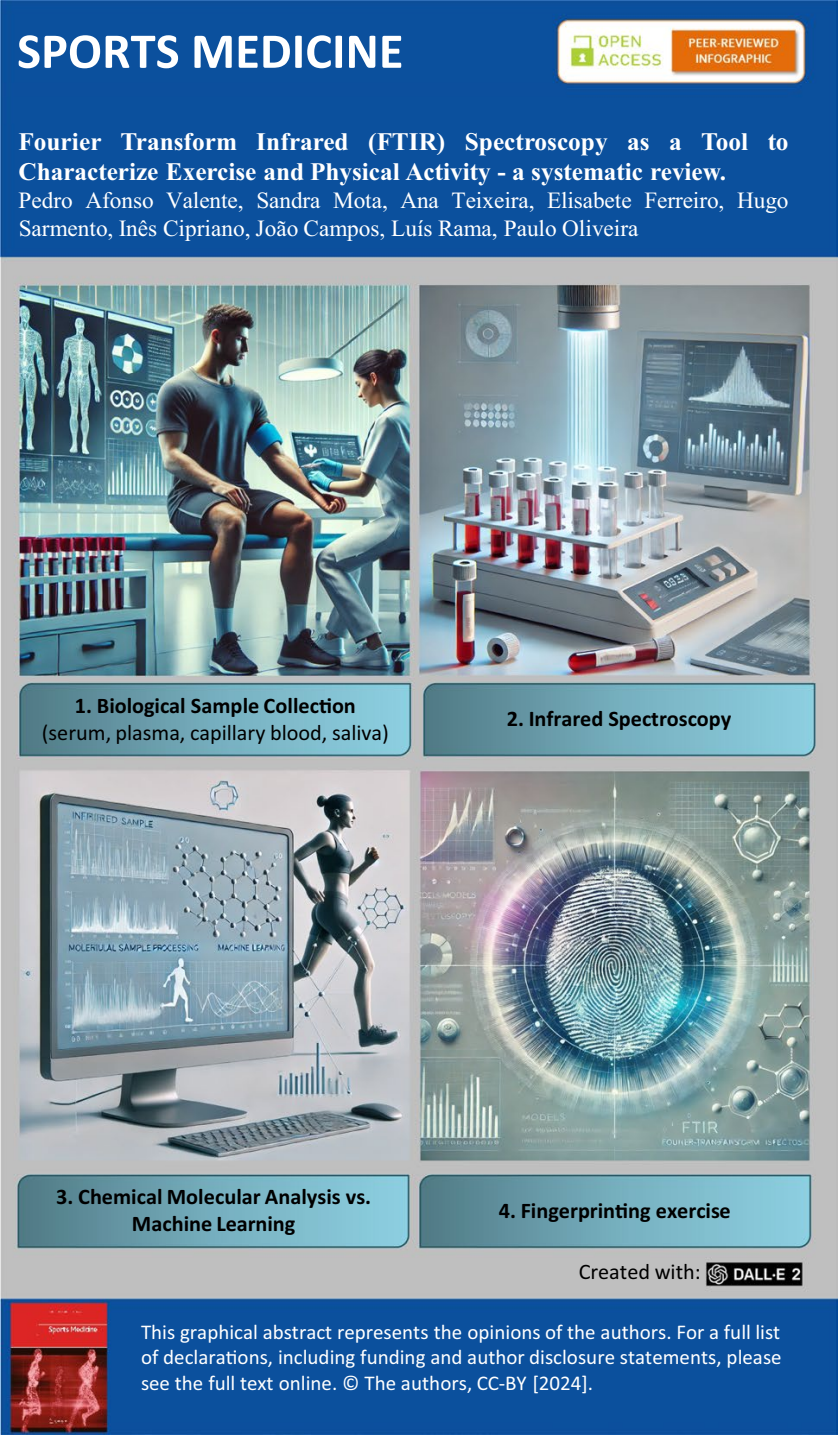

## Key Points

**Table:** 

It is possible to identify certain molecular groups and understand how they respond to exercise, using Fourier transform infrared spectroscopy.
Fourier transform infrared spectroscopy presents an opportunity to be considered a potential biological fingerprinting method.
This technique may provide valuable insights into how athletes respond to exercise, preventing fatigue and improving performance.

## Introduction

Since the mid-twentieth century, the scientific community has increasingly highlighted the significant societal benefits of physical activity. In tandem, research teams have focused on developing innovative methods to analyze the physiological and metabolic responses to exercise. Beyond health promotion, the economic impact of physical inactivity is substantial; Santos et al. estimate a global cost of approximately £47.6 billion annually. [1]. Furthermore, sports sciences have honed their focus on elevating athletic performance, deploying strategies to push the boundaries of physical prowess, prevent injuries, and optimize athlete potential. While it is widely acknowledged that challenging the body’s limits is essential for maximizing gains, navigating the fine line between this necessity and chronic fatigue remains a delicate task [2]. Accurately pinpointing the precise physical peak that may prove detrimental to an athlete is still a formidable challenge.

To understand the effects of training, researchers are investigating novel biomarkers based on the identification of previously unknown metabolic pathways influenced by physical activity. Currently, there are no established biomarkers specifically associated with overtraining, as the exact cause of overtraining is not fully understood, and there is no universal tool to predict its occurrence before it is clinically diagnosed [3].

In addition to these existing biomarkers, researchers also scrutinized heart rate responses to exercise and changes in oxygen and carbon dioxide levels during inhalation and exhalation, shedding limited light on the comprehensive metabolic landscape [4]. Of note, these methodologies are truly invasive, and participants are never motivated to agree to them repeatedly.

In response to the above-mentioned limitations, Fourier transform infrared spectroscopy (FTIR) has emerged as a relevant tool for a simultaneous multi-component analysis. Fourier transform infrared spectroscopy, a vibrational spectroscopy technique, stands out for its speed and accuracy, which contrasts with conventional methods. At its core, FTIR explores the periodic vibrations of covalently bound atoms within chemical compounds, discerning between stretching and bending vibrational modes, further subdivided into symmetric and anti-symmetric stretching and various bending modes, enabling the study of the role of the surrounding environment on protein conformation [5]. Each molecule exhibits a unique and distinctive vibrational signature, resulting in a spectral “fingerprint” for each compound in the sample [6]. However, translating this chemical potential into the realm of biological samples has proven arduous. The scientific community continues to grapple with this intricate challenge. Yet, progress has been made over the past five decades, primarily attributed to two pivotal factors: the improvement and increased accessibility of spectrometers and the evolution of computational capabilities, which enable the analysis of complex data and the extraction of new insights [7].

Beyond its sensitivity and accuracy, FTIR offers several advantages over alternative techniques. It boasts high reproducibility and requires minimal sample quantities—typically a few sample microliters suffice for analysis. Notably, FTIR does not necessitate intricate sample preparation procedures, preserving the integrity of the samples. Nonetheless, certain disadvantages, if unaddressed, can hinder the technique’s success. Thus, FTIR spectroscopy encounters two principal challenges that limit spectral accuracy. First, the presence of water molecules within samples introduces interference, as water’s specific vibrational modes strongly absorb infrared radiation. Consequently, water signals often overshadow signals from other target components. To mitigate this interference, researchers frequently employ desiccation methods, reducing water content and enhancing the clarity and accuracy of characterizing other sample constituents [7]. Second, the data analysis is particularly complex. Yet, with developments in technology, it seems clear that this problem will become easier to solve.

In summary, FTIR spectroscopy presents both immense promise and specific challenges. Its application holds tremendous potential in fields such as disease detection and prediction or even for the analysis of biopharmaceuticals [8], particularly when coupled with machine-learning models. Its is recognized that infrared fingerprinting can already detect and distinguish various common cancer types [9]. Importantly, the use of FTIR spectroscopy in sports medicine has been explored, but its complete validation is still underway. Thus, this systematic review comprehensively evaluates the body of research employing FTIR to investigate biological samples, encompassing individuals spanning from recreational enthusiasts to elite athletes. Our aim is to substantiate the potential of FTIR spectroscopy to elucidate the impacts of exercise and physical activity on diverse biomarkers, thereby contributing to the growing body of knowledge in exercise science and biomarker research.

## Methods

This systematic review adhered to the PRISMA (Preferred Reporting Items for Systematic reviews and Meta-analyses) guidelines [10]. The study protocol was registered in the International Prospective Register of Systematic Reviews (PROSPERO) under the ID number CRD42023441965. The analysis was independently conducted on 28 March, 2023. This protocol can be accessed via the website: https://www.crd.york.ac.uk/prospero/display_record.php?RecordID=441965 (Accessed 30 November, 2023).

### Eligibility Criteria

Inclusion criteria for the articles encompassed: (1) original papers in English; (2) studies examining or evaluating exercise or physical activity; (3) experiments conducted on humans aged 18–50 years (in vivo experiments) with no exclusions based on chosen lifestyle; (4) collection of biological samples (e.g., urine, saliva, capillary or venous blood); and (5) employment of FTIR spectroscopy with no exclusion for the mathematical analysis used. Studies related to subjects with chronic pathologies or acute injuries and nutritional effects of compounds, and those devoid of relevant data or conference abstracts were excluded. Given the absence of prior systematic reviews on this topic, we imposed no time limitations.

### Information Sources

Four databases were searched: PubMed; Scopus; Web of Science, and SPORTDiscus. The searches were performed on 28 March, 2023. Additionally, a manual search was performed on the study’s references to identify potentially interesting papers that could complement this review’s discussion.

### Search Strategy

For the search process, Boolean operators were used, such as AND/OR and “*”. We used keywords such as “FTIR” OR “Fourier transform infrared” combined with “Spectroscopy” OR “Spectrum Analysis” and further combined with “sport*” OR “exercise*” OR “physical activity” OR “athlete*” in the “all fields” category.

### Selection Process

The retrieved records (title, abstracts, and full texts) were independently screened by two of the authors (PV and IC). In instances of disagreement between reviewers, on article inclusion, the final decision rested with the senior author (HS), recognized as an expert in this field. The EndNote TM X 9.3.3 software (Clarivate TM) was used to manage the records, including the removal of duplicates, either automatically or manually.

### Data Extraction Process and Data Items

The data from the studies were collected by two authors (PV, IC) and shared in a Google sheet to organize the data. Data were organized considering the publication year, sample categorization (age and sex), and sample type (e.g., venous or capillary blood, or saliva). We also considered the type of exercise and the designed protocol, i.e., which type of exercise was studied, if an exercise protocol was designed for the sample collection (acute response to exercise), or if it was performed in the absence of previous specific exercise (chronic response to exercise). Moreover, the type of spectra analysis (chemical molecular spectra analysis) and the results analysis (mathematical model to predict any pattern or regular laboratory methodologies to compare with the spectra peaks) were also taken into consideration.

### Study Risk of Bias Assessment

The methodological quality of the studies was evaluated using the Quality Assessment Tool for Observational Cohort and Cross-Sectional Studies, developed by the National Heart, Lung, and Blood Institute (updated July 2021) [11]. This scale was divided into 14 different questions: 1. Was the research question or objective in this paper clearly stated? 2. Was the study population clearly specified and defined? 3. Was the participation rate of eligible persons at least 50%? 4. Were all the subjects selected or recruited from the same or similar populations (including the same time period)? Were inclusion and exclusion criteria for being in the study prespecified and applied uniformly to all participants? 5. Was a sample size justification, power description, or variance and effect estimates provided? 6. For the analyses in this paper, were the exposure(s) of interest measured prior to the outcome(s) being measured? 7. Was the timeframe sufficient so that one could reasonably expect to see an association between exposure and outcome if it existed? 8. For exposures that can vary in amount or level, did the study examine different levels of the exposure as related to the outcome (e.g., categories of exposure, or exposure measured as continuous variable)? 9. Were the exposure measures (independent variables) clearly defined, valid, reliable, and implemented consistently across all study participants? 10. Was the exposure(s) assessed more than once over time? 11. Were the outcome measures (dependent variables) clearly defined, valid, reliable, and implemented consistently across all study participants? 12. Were the outcome assessors blinded to the exposure status of participants? 13. Was loss to follow-up after baseline 20% or less? 14. Were key potential confounding variables measured and adjusted statistically for their impact on the relationship between exposure(s) and outcome(s)? All the questions were answered with 1 (Yes), 0 (No) or n.a. (not applicable), and a final percentage was calculated. Studies were classified as good (over 85% quality rating), fair (between 50 and 85%), or poor (under 50%).

Two independent reviewers performed the quality assessment. In cases of disagreement, a consensus was reached through discussion or with the assistance of a third author of this study.

## Results

### Search, Selection, and Inclusion of Publications

**Fig. 1:**
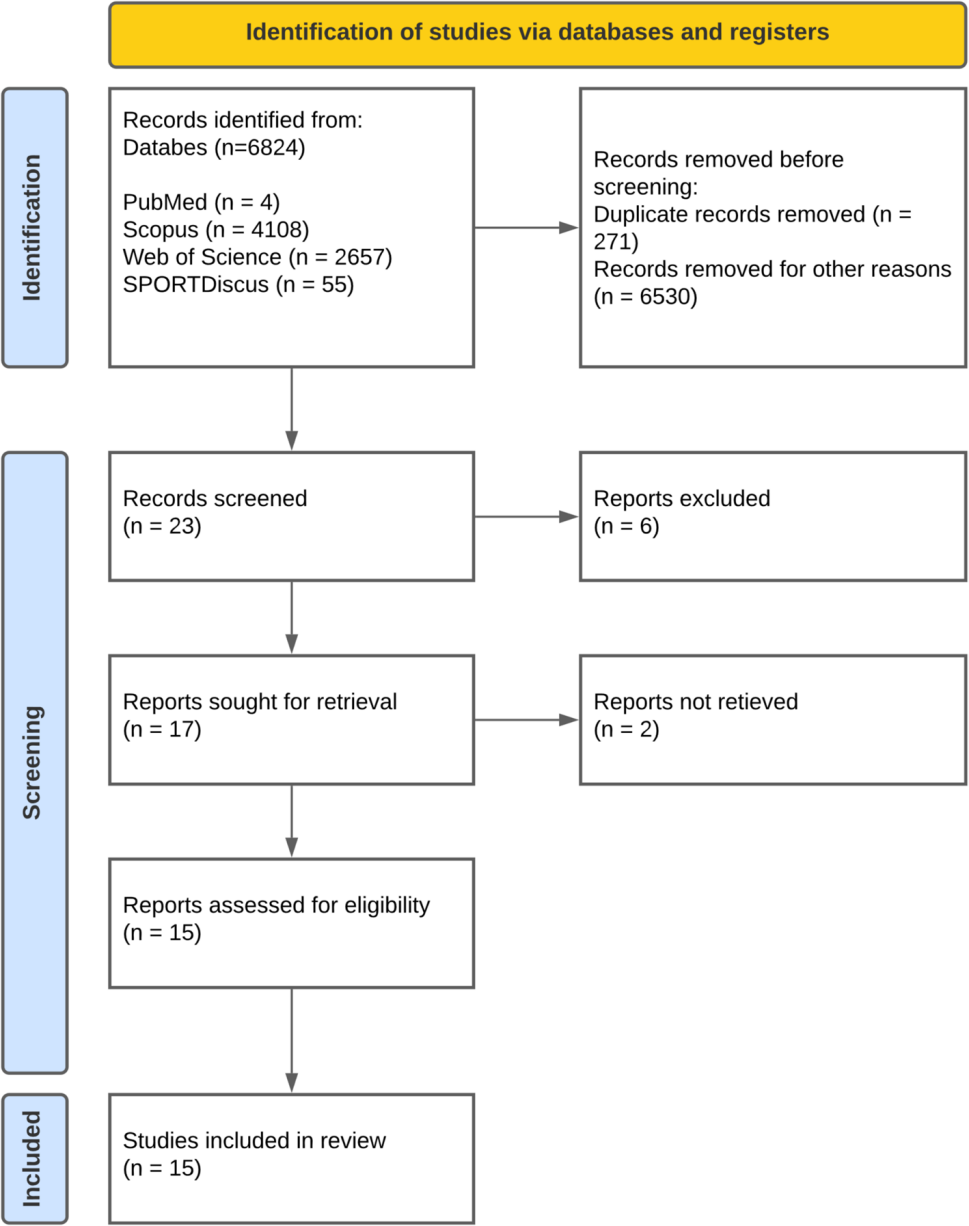
Flowchart of the procedures used for the article search

We initially identified 6824 references across the four databases. No additional references were added subsequently. Following duplicate removal (271 references) and title screening (6,530 studies), we excluded an additional six studies after assessing abstracts. Of the remaining 17 references, two could not be accessed in full text and the authors did not give access to the papers. Upon a comprehensive text analysis, we found no further grounds for exclusion, resulting in 15 studies eligible for further examination and analysis [12, 13] (Fig. 1). Notably, 50% of the selected papers were authored by Petibois and Déléris [2, 14–20], who also spearheaded the inaugural paper on this topic in 2000 [2]. Moreover, to the best of our knowledge, no previous systematic reviews had been conducted on this subject, and half of the studies were published between 2000 and 2005.

### Quality of the Studies

Regarding study quality, four studies achieved ratings exceeding 80% (considered good) [2, 21–23], while 11 studies fell within the 50–80% range. No study received a negative rating, resulting in the inclusion of all studies in this systematic review. Noteworthy is that two studies [21, 23] received the highest rating (Table 1).

**Table 1:** Quality assessment of each included systematic review

	[2]	[14]	[15]	[16]	[17]	[18]	[19]	[20]	[21]	[26]	[37]	[24]	[25]	[22]	[23]
Q1	1	0	1	1	1	1	1	1	1	1	1	1	1	1	1
Q2	1	1	1	1	1	1	1	0	1	1	1	1	1	1	1
Q3	1	1	1	1	1	1	1	1	1	1	1	1	1	1	1
Q4	1	1	0	1	1	1	1	0	1	1	1	1	1	1	1
Q5	0	0	0	0	0	0	0	0	1	0	0	0	0	0	1
Q6	1	1	0	0	0	0	0	1	1	1	1	1	0	1	1
Q7	1	0	1	1	0	1	1	1	1	1	1	0	0	1	1
Q8	1	0	n.a	0	1	0	0	1	1	1	0	0	n.a	1	1
Q9	1	1	1	1	1	1	1	1	1	0	0	1	1	1	1
Q10	1	1	1	1	0	1	1	1	1	0	1	1	0	1	1
Q11	n.a	1	1	1	1	1	1	1	1	1	1	1	1	1	1
Q12	1	1	1	n.a	1	n.a	n.a	1	1	1	1	1	1	1	1
Q13	1	1	1	1	1	1	1	1	1	1	1	1	1	1	1
Q14	1	1	1	1	1	1	1	1	1	1	1	1	1	1	1
**Total (%)**	**92**	**71**	**77**	**77**	**71**	**77**	**77**	**79**	**100**	**79**	**79**	**79**	**69**	**93**	**100**

*n.a.* not applicable, *Q* question

### Demographic and Biological Characterization

Out of the 15 analysed studies, all exclusively enrolled male participants, which is a major limitation. Among these references, four concentrated on rowing athletes or former rowing athletes, while four simultaneously examined multiple sports at the same time (e.g., football, basketball, tennis, Muay Thai, karate, boxing, and long-distance running). Two studies followed handball protocols, and two others focused on rugby athletes. Cyclists, swimmers, or marathon runners formed the subject of the other studies. Urine samples were not collected in any of these studies. Instead, nine studies used capillary blood, three utilized venous blood, and six chose saliva for analysis. A saliva analysis gained prominence with its introduction in a 2010 study [21]. Regarding the study design, four studies analyzed long-term periods with multiple exercise sessions, while 11 studies scrutinized acute metabolic and biological responses to exercise. The most recent study examining chronic exercise responses was published in 2005 [18].

### Biological Sample Analysis

Three categories of biochemical and chemical analyses were identified among the 15 studies: FTIR for molecule identification, FTIR combined with machine-learning algorithms, and a traditional biochemical laboratory analysis. Eleven studies employed FTIR spectra for molecule identification, including proteins. Machine learning or big data analysis was applied in seven studies using various techniques. Notably, only three studies compared these newer analyses with traditional laboratory methods commonly used in the scientific community [21, 24, 25]. Multiple studies analyzed spectra in different regions, with a particular focus on =CH (CH_2_ and CH_3_) and N–H bands, followed by C–O and C=O bands. Additionally, ten other region bands received analysis attention, albeit in a limited number of references (Table 2).

**Table 2:** Study characterization

Study	Sample characterization	Biological samples	Type of exercise and sample collection protocol	Biomolecular analysis
[2]	20 elite rowers	Capillary blood	Over 37 weeks (10 times per week), samples collected before and after each session	FTIR spectra acquisitions to calculate FAE, TG, AM I, and AM II; Ward’s method for spectra classification; clustering analysis
[14]	15 elite rowers	Capillary blood; venous blood	Over 5 weeks, 3rd, 5th, and 7th week (samples collected at rest and after each training session)	Capillary blood: FTIR spectra; venous blood: plasma Gl, Lac, TP and Alb, FAM, TG, and AA
[15]	14 top-class marathon runners	Capillary blood	10-km run, samples collected before and after the end of the race	FTIR spectra to calculate serum Gl, Lac, U, TG, glycerol and major protein levels
[16]	13 elite rowers	Capillary blood	Over 8 weeks and in weeks 10, 13, 15, 18, 20, 23, 28, 33, 37, 41, and 47, samples collected before and after each training session	FTIR spectra to measure Gl, Lac, U, Alb, haptoglobin, transferrin, ⍺1-acid glycoprotein, ⍺2-macroglobulin, ⍺1-antitrypsin, IgA, IgD, IgG1, IgG2, IgG3, IgG4, and IgM, Apo-A1, Apo-B, and Apo-C3
[17]	7 national level swimmers	Capillary blood	Samples collected before and at the end each swimming test, 400 m, 300 m, 200 m, and 100 m	FTIR spectra to measure Gl, Lac, U, Alb, haptoglobin, transferrin, ⍺1-acid glycoprotein, ⍺2-macroglobulin, ⍺1-antitrypsin, Apo-A1, Apo-B, and Apo-C3
[18]	15 previously endurance rowers	Capillary blood	19 weeks of training period, samples collected before and after the training session, once per week	FTIR spectra to calculate Gl, phosphate Lac, U, FAM and AA, EMC
[19]	21 trained subjects	Capillary blood	120 min cycling exercise, samples collected at rest and every 20 min	FTIR spectra to calculate plasma HC
[20]	16 trained cyclists	Capillary blood	2 h exercise, collected at rest and every 15 min	FTIR spectra to calculate Gl, Lac, glycerol, TG, Alb, FAM, and plasma volume changes
[21]	48 well-trained athletes	Venous blood and saliva	Ramp test on a treadmill, samples collected before the beginning, after the end and after 30 min	Salivary assays used to collect STPC, salivary sIgA and cortisol, ⍺ amylase activity, phosphates, and U; serum: CK, As AT, AlAT, and GH
[26]	14 professional handball athletes	Saliva	Samples collected before, immediately after and 2 h after the end of a simulated handball match	n.a
[37]	13 rugby players	Saliva	Samples collected at 7.00 a.m., before and immediately after a fatigue test treadmill	n.a
[24]	10 rugby players	Venous blood	Samples collected 30 min before the training session, immediately after and 24 h after the training session	Serum cortisol
[25]	14 professional handball athletes	Saliva collection	Samples collected before the match warm-up, 5 and 120 min after the end of a match	FTIR spectra acquisition sIgA and cortisol
[22]	6 amateur athletes	Saliva and capillary blood	Maximal progressive stress test on a treadmill, samples collected before and at the end of each completed stage of the maximal progressive test	n.a
[23]	57 athletes	Saliva	Samples collected after the physical activity (1 training session)	n.a

*AA* amino acids, *Alb* albumin, *Al AT* alanine aminotransferase, *AM* amide, *Ap* apolipoproteins, *As AT* aspartate aminotransferase, *CK* creatine kinase, *EMC* erythrocyte molecular content, *FAE* fatty acids esters, *FAM* fatty acyl moieties, *GH* growth hormone, *Gl* glucose, *h* hours, *HC* hemo concentration, *Ig* immunoglobulins, *Lac* lactate, *min* minutes, *Ref* reference, *STPC* salivary total protein concentration, *TG* triglycerides, *TP* total proteins, *U* urea

## Discussion

### Chemical Spectral Analysis

The majority of investigations into a spectral analysis have traditionally adhered to a chemical and classical framework, wherein the focus has been on the examination of vibrational modes to discern distinct molecular components. Notable vibrational segments under scrutiny encompass CH_2_ (associated with lipids) [2, 14, 15, 19–21, 25, 26]; CH_3_ (also related to lipids) [2, 14, 15, 19–21, 25, 26]; and groups that characterize proteins such as C=O (representative of amide I) [2, 14, 15, 18, 19, 21, 25]; and N–H (associated with amide II) stretching or bending modes [2, 14, 15, 21, 26] (Table 2). For the first time in athletes’ capillary blood, Petibois et al. conducted a comprehensive analysis of over 20 peaks associated with various macromolecules and proteins, including glucose, lactate, urea, albumin, haptoglobin, transferrin, α1-acid glycoprotein, α2-macroglobulin, α1-antitrypsin, several immunoglobulins (IgA, IgD, Ig1, Ig2, Ig3, Ig4), and apolipoproteins using infrared spectroscopy [16, 17]. Along the same lines, Khaustova et al. successfully analyzed total protein, sIgA, and cortisol levels in saliva of athletes [21]. Of note, the analysis of other molecular groups showed no significant results [21, 24, 25, 27] (Table 3).

**Table 3:** Study results and conclusions

Study	Aim	Machine learning	Conclusions
[2]	Use different IR spectra, evaluate the metabolic response to standardized endurance training	Clustering	Difference between over-reaching and overtraining: peptide alteration (amide I) in response to endurance exercise
[14]	Use FTIR spectrometry for plasma levels of FAM. Correlate known blood biomolecular changes during endurance exercise with plasma FTIR spectra	n.a	Plasma FTIR spectroscopy describes the global metabolic response to metabolic stress (e.g., endurance exercise); of FAM and AA levels accurately determined on FTIR plasma spectra by using their most characteristic absorbances
[15]	Determine which metabolic parameters may explain the shift toward 127 min, from 142 min, finish a marathon	n.a	Best performance, metabolic response to exercise parameters: higher Gl level increase; less unsaturated blood fatty acids; more pronounced decrease in blood TG content concomitantly to a higher glycerol level increase; higher AA production and blood release; possible catabolism of several proteins for AA supply to skeletal muscles
[16]	Describe training effects on exercise-induced changes in blood content	n.a	Indicator for skeletal muscle protein turnover during training: changes in α1-AGp levels. Understanding immune response to exercise: analysis of IgA subclasses providing a better understanding than total IgG levels
[17]	Determine blood content changes during a 400-m swimming test	n.a	Greater blood glucose increase at exercise; inverse pattern in Lac level; larger change in Lac level at the end of exercise, suggesting higher glycolysis utilization; higher fatty acids availability and selectivity
[18]	Provide direct evidence that endurance training induces biochemical adaptations within erythrocytes against high OS of acute exercise	n.a	Hard endurance training induced specific cellular adaptations, linked with a decrease in OS effects on erythrocyte during exercise; long-term endurance training can lead to a future atherosclerotic profile
[19]	Use FT-IR applications for determining OS effects on erythrocytes during an intensity endurance exercise	n.a	Phospholipid alteration measured by kinetic changes of v = (CH), vas(CH3), vas(CH2), and vP=O absorbances; peroxidation level determined with the CH2/CH3 and C=H/CH3 ratios. Hb denaturation during OS observed through a biphasic pattern of changes for v(C = O) and o(N–H) absorbances; C=O/N–H increase related to Hb unfolding and linked to O2 flux level, and independent v(C=O) absorbance increase; significant Hb carbonylation while cell acidosis and dehydration increased
[20]	Analyze changes in plasma FAM (leveL and chain characteristics)	n.a	Opposite changes between carbohydrate and fatty acid metabolism: decrease of glucose and Lac levels; increased albumin, FAM, and TG during the second hour of exercise. Pattern not observed after the end of exercise
[21]	Apply and modify ATR FTIR spectroscopy method for calculating α-amylase, cortisol, salivary IgA, U, TP, and phosphate levels in saliva. Monitor metabolic changes in athletes’ saliva composition during short-term HIT using pre-established calibration models	PLS	Correlation between the salivary biomarker levels and the FTIR spectral analysis; possible to use FTIR for the saliva analysis (saliva TP, cortisol, α-amylase, salivary IgA, U, and phosphate). Use of patterns of cortisol, α-amylase, and secretory IgA level changes during short-term high-intensity exercises to check real-time response to stress
[26]	Show the main biochemical differences between saliva phases, supernatant, and precipitate, as well as the importance of these phases to better classify the physiological stress in athletes by FT-IR	PCA and LDA Ward’s algorithm for cluster analysis	Both phases of saliva discriminate the FTIR spectra
[37]	Discriminate FT-IR spectra of mixed saliva collected before and after the fatigue test continued	Cluster analysis	Division of athletes into three different groups using saliva samples and cluster analysis
[24]	Examine the impact of training sessions on the level of serum cortisol in rugby players	n.a	FTIR spectroscopy to analyse exercise; no increase on cortisol levels during exercise
[25]	Assess isokinetic parameters of hamstring and quadriceps muscles in male handball players, alongside salivary cortisol and sIgA (using FT-IR)	n.a	No significant spectral differences to saliva sample collected pre-match and post-match, as well as after 2 h of recovery
[22]	Investigate the biochemical changes in saliva using infrared spectroscopy during maximal stress test	PCA, PC-LDA, LOOCV	Salivary Lac is higher in sprinters after a 400-m run when compared with long-distance runners; FTIR is an accurate, non-invasive, and trustable method to analyse Lac and Gl levels
[23]	Develop an easy-to-follow, fast, non-invasive, and reliable ATR-FTIR-based method for analyzing saliva samples to qualitatively determine the biochemical profile post-physical exercise	PCA, PLS-DA	Development of a non-invasive and predative method for the athletes’ status classification

*AA* amino acids, *AGp* acid glycoprotein, *D* discriminant analysis, *FAM* fatty acyl moieties, *Gl* glucose, *h* hours, *Hb* hemoglobin, *HIT* high-intensity training, *Ig* immunoglobulins, *Lac* lactate, *LDA* linear discriminant analysis, *LOOCV* linear one out cross-validation, *OS* oxidative stress, *min* minutes, *n.a.* not applicable, *PCA* principal component analysis, *PLS* partial least square method, *TG* triglycerides, *U* urea

#### CH_2_: Long-Chain Fatty Acids and Phospholipids, and CH_3_: Cholesterol Esters, Triglycerides, and Glycerol

The CH_2_ band, which divides into two peaks (symmetric [2950–2880 cm^−1^ and asymmetric [2870–2830 cm^−1^]), can be associated with various compounds, including long-chain fatty acids and phospholipids. Importantly, Petibois and colleagues attempted to understand how a specific molecular group responds to exercise in athletes over various durations [2]. Initially, they directly analyzed how to utilize this peak for diagnosing fatigue. Unfortunately, no conclusive results were obtained from this analysis [2]. Similar observations were made with the CH_3_ group [2880–2950 cm^−1^ and asymmetric [2880–2860 cm^−1^]). However, they did ascertain that these molecular spectra indeed react to exercise. Consequently, a few years later, they employed this technique to assess the fatty acyl moieties (FAM) before and after exercise. They focused on studying FAM instead of free fatty acids because the former, when found in plasma, may undergo metabolism under conditions of metabolic stress by free fatty acids. To achieve this, they pinpointed a more precise peak 2996–2819 cm^−1^. For the first time, they successfully established a direct link between FTIR spectra and plasma FAM measurements in athletes [14]. This study not only examined the FAM level but also analyzed v(COO¯) [spectral area 1430–1360 cm^−1^) and its correlation with the amino acid level. The results notably demonstrated how this analysis aids in the easy identification of new biological responses to exercise. Subsequently, several other studies by Petibois and his team pursued a similar scientific approach, primarily utilizing this technique to better comprehend how CH_2_ and CH_3_ respond to exercise [15, 18–20].

In conclusion, this technique enables specific analyses that would otherwise be unfeasible through conventional means. When assessing the acute response to exercise, it becomes possible to scrutinize multiple biomarkers with a simpler collection process. For instance, in the latest study published by Petibois and Déléris [20], in which they evaluated the acute response to a 2-h cycling exercise, they discerned an inverse correlation between carbohydrate and fatty acyl metabolism. Specifically, a decrease in glucose and lactate levels was observed alongside an increase in albumin, FAM, and triglyceride levels [20]. Such analyses are only achievable through spectroscopy by simultaneously analyzing different peaks.

#### C=O: Lipids, Cholesterol Esters, Triglycerides, and Amide I

The bands in the C=O region can be categorized into stretching vibrations and bending vibrations of the molecule, and each is associated with different aspects. Stretching is related to lipids and cholesterol esters and can be identified in the wave number range of 1739–1732. Bending is associated with amide I, proteins, and amino acids, and is recognized in the wave number range of 1720–1600 cm^−1^. Petibois et al. pursued the previously described path focusing on the C=O band, specifically targeting the biomarkers it might represent. However, establishing a direct link between this peak and the acute response to exercise, both before and after the designed cycling protocol, proved challenging [14].

Several years later, Caetano Júnior et al. also endeavored to study this molecular vibrational band along with others such as C–O, C–C, and P=O, in relation to changes in salivary cortisol and sIgA [25]. Through this study, the investigators pinpointed the principal FTIR region for measuring sIgA and cortisol –CH_2_/CH_3_, C=O, COO¯, and P=O, with a range between 1484 ${\text{cm}}^{-1}$ and 1191 ${\text{cm}}^{-1}$. This investigation did not solely focus on this band region but also scrutinized C–O (stretching), C–C, C–O–H (angular bending), and P=O (symmetric stretching vibrations) linked with the cortisol molecule. However, there were no discernible differential spectra observed before and after the conclusion of the handball match, despite analyzing both regions. Nevertheless, correlating the cortisol fluctuations with the applied training loads made it possible to identify positions requiring greater stress during the match [25].

This underscores the precision and minute nature of this analysis. It raises questions about why the spectra do not respond to acute exercise, considering the myriad effects that exercise has on biological biomarkers and the technique’s inherent accuracy.

#### N–H: Amide II, α-Helix Proteins, and Amino Acids

The N–H band refers to the bending vibration, associated with the wave number range of 1600–1480 ${\text{cm}}^{-1}$, which serves as a marker for amide II or α-helix proteins and amino acids. This vibrational band has been extensively employed by Petibois et al. [18]. In this scientific investigation, the team also examined the relationship between this specific molecular group and the diagnosis of overtraining. However, the results mirrored those obtained previously. Conversely, while analyzing other molecular groups, establishing a reasonable connection with certain biomarkers was relatively straightforward. However, in this particular case, there was a lack of clarity. Nonetheless, this ambiguity can still contribute to the analyses. For instance, in a study aiming to furnish direct evidence that endurance training prompts biochemical adaptations within erythrocytes to counteract the high oxidative stress induced by acute exercise, capillary blood was collected from a group of cyclists over a 12-week training period. The study assessed the superoxide dismutase activity following the classic methodology outlined by McCord and Fridovich in 1969 [28]. Within this study, the N–H absorption region (bending vibrations) demonstrated a strong correlation with the stretching of C–(H)_n_ absorption in phospholipids and fatty acids, as well as stretching C=O. Notably, a conspicuous increase in this pattern was observed after the tenth week of exercise [18]. This observation allows for a better understanding of the biochemical changes induced in erythrocytes by exercise and sheds light on the potential associations of the N–H group with other molecular entities. Furthermore, it is crucial to acknowledge the diverse range of conclusions that can be drawn using this method.

#### Summary

Overall, studies demonstrated that FTIR has enabled the identification of specific molecular moieties and proteins within salivary and blood samples, with precision. Remarkably, FTIR-associated peaks highly correlate with molecule levels obtained by conventional laboratory protocols [21, 27]. However, FTIR alone has not provided clear evidence of significant biological responses, resulting in inconclusive findings. At the same time, it is important to recognize the large volume of data that can be easily collected with this technique, which can turn this limitation into an opportunity. Overall, these points highlight the considerable potential of FTIR to enhance our understanding of biochemical adaptations to exercise.

### Data Analysis Using Different Mathematical Models

In 1991, Helm et al. [29] demonstrated that spectroscopy could be complemented using various mathematical models. To the best of our knowledge, their study represented one of the first instances in which spectral data were employed as a fingerprinted pattern for characterizing biological substances. They showed that bacterial FTIR spectra can have fingerprint-like patterns, resulting from the superimposed absorbance bands of all constituents of the cell [29]. Their analysis successfully identified previously unknown bacterial species through clustering and showcased the method’s potential for a highly detailed analysis (Table 3).

Nine years later, Petibois et al. conducted a study on FTIR and sports metabolism, marking the first connection between these two fields [2]. The study aimed to assess the relationship between metabolic responses and endurance training sessions by evaluating several spectral regions, including N–H, C=O, CH_2,_ CH_3_, =CH, and C–O, over a period of 37 weeks (once per week), in capillary blood. However, the scientific team did not conduct a detailed chemical analysis of the obtained peaks. Instead, with the support of a clustering analysis, they automatically grouped the athletes based on the heterogeneity of each spectrum.

By week 7, it became possible to separate the athletes into three main groups, denoted as Group A, B, and C. The primary difference observed was in the C–O absorption region, associated with saccharide absorption. These differences correlated with the performance of each group, with Group A showing significantly lower performance compared with the other two groups. At week 8, differences were also confirmed in the =CH absorption region, related to cholesterol ester absorption, following a similar trend. However, the most significant finding emerged in week 15, when a division was observed in the N–H absorption region, specifically related to amide II. The results evidenced that athletes clinically diagnosed with overtraining presented a higher N–H absorption peak. This study introduced the idea that the overtraining process is not primarily driven by peptide and amino acid metabolism, but rather by changes in sugar and lipid metabolism. Even so, these peptide and amino acid observed alterations are the outcome of overtraining or over-reaching processes [2].

By using saliva, Khaustova et al. also made a significant advancement in this field, establishing a correlation between biomarker levels and spectral peaks through various mathematical models [21]. This research team conducted an extensive analysis, involving more than 120 samples of athletes for calibration and 34 spectra for validating a new partial least square (PLS) model. In addition to this, the team also determined salivary total protein, sIgA, cortisol, and α-amylase levels in saliva using conventional methods for all the athletes that were participating. The developed models exhibited a high predictive capability with the following results: protein level (spectral regions: 1503–1440, 1317–1249, 1190–936): = 0.94); sIgA (spectral regions: 1567–1526, 1488–1406: = 0.86); cortisol (spectral regions: 3500–3200, 1900–900: = 0.90); and α-amylase (spectral regions: 1578–1548, 1526–1496 1444–1305: = 0.91). These results underscore the simplicity and accuracy of this method, making it applicable to athlete training control and the analysis of large-scale population lifestyles.

In another study, Chrimatopoulos et al. remarkably demonstrated the ability to assess the physical condition of athletes using only saliva samples and FTIR [23]. They employed a PLS discriminant analysis (DA) to construct a multi-sport analysis model. The initial phase of the study involved measuring a high-level athlete to gain insights into the biochemical changes during exercise. Subsequently, the study evaluated 56 athletes simultaneously, comprising 19 low-level athletes and 13 high-level athletes. Out of these, 42 athletes were utilized for dataset development, while the remaining 14 athletes were reserved for testing. For the development of the model that would be able to characterize the sample, the following IR bands were used: 1196 ${\text{cm}}^{-1},1529 {\text{cm}}^{-1},$ and 1642 ${\text{cm}}^{-1}$ for PC1 and 1039 ${\text{cm}}^{-1}, 1543 {\text{cm}}^{-1}$, and 1619 ${\text{cm}}^{-1}$ for PC2, representing glucose, glycogen, sugar moieties, amide II, and amide I, respectively. Both the training and testing datasets were constructed using FTIR spectra, with specific bands selected for this purpose. Notably, the developed model achieved a remarkable accuracy of 98%, successfully distinguishing between low-level and high-level athletes [23].

In conclusion and considering all the studies analyzed and the conclusions taken from each, it is possible to understand the mathematical models that are able to achieve a more accurate and trustable machine-learning model or more relevant outcomes. There are definitely great improvements on data treatment methods with a clustering analysis, a principal component analysis, and PLS-DA emerging as promising techniques. These recent studies have achieved high sensitivities, suggesting the potential for further improvement [2, 15, 21].

### Applications and Limitations of the Technique

As mentioned previously, the first study connecting FTIR to physical activity was published in 2000, and since then, 15 studies have linked infrared spectroscopy and exercise evaluation. The applications of this technique are extensive. For example, FTIR has been used to identify metabolic and biochemical differences between overtraining and over-reaching, even predicting the early stages of overtraining [3]. It has also been applied to analyze acute and chronic responses of specific metabolites to exercise, as well as to differentiate athletes based on their metabolic capacity. Interestingly, Petibois and Déléris also identified two major drawbacks of this methodology: the inability to operate in aqueous environments because of water absorption and data treatment complexity [30]. However, recent developments suggest that these barriers may eventually be overcome, with novel techniques, spectrometers, and software applications currently in development [30].

Aqueous environment-associated limitations can be circumvented by removing the water spectra through the application of a new technology—an automated FTIR device with a flow-through transmission cuvette (CaF2 with 7–10 μm path length), instead of the traditional method of dehydrating the sample [6]. This was a significant step-forward in this field. Second, data treatment is also becoming more resourceful with the new mathematical models (with the support of machine-learning systems) that are being developed and with the help of new ultra-efficient computers [9]. This paper was innovative as it substantiated the feasibility of mitigating biological variability within individual subjects through the utilization of infrared spectroscopy and underscored the possibility of leveraging hydrated samples, such as plasma or serum, for the development of an infrared spectrum [6, 9].

Other limitations of the studies published in the field are related to the study design. It is imperative to acknowledge that sample size plays a pivotal role in shaping the robustness of predictive models, suggesting that larger sample sizes could potentially augment the capacity for finer distinctions between athletes [15]. Importantly, research dedicated to acute exercise responses involved sample sizes of up to 57 individuals, and investigations on long-term exercise adaptations included a maximum of 20 athletes [2]. For comparison, a study in which FTIR spectroscopy was applied to early-stage cancer prediction encompassed a cohort of 200 individuals [9, 31].

Moreover, the absence of female participants limits the generalizability of findings. Therefore, based on results from male participants and to ensure studies include both sexes, this technology should be tested also on female individuals. Furthermore, there are no studies working directly with recreational athletes or even with sedentary people. The studies focused exclusively on high-level or elite athletes, without addressing broader health-related purposes for the general population. The biological samples used in these studies can also be a limitation. Thus, in certain scientific disciplines, such as those employing infrared spectroscopy, urine and semen have already been successfully used [32, 33]. It is clear that incorporating these types of samples into the research framework could improve the effectiveness of model development and potentially lead to a shift in laboratory sample collection methods.

## Conclusions

Fourier transform infrared spectroscopy holds significant promise as a valuable tool for analyzing, prescribing, and monitoring exercise in the near future. It has the potential to offer unique insights previously unattainable through other methods. The technique’s accuracy is suitable for monitoring and enhancing the performance of high-level athletes, aiding in preventing overtraining and assessing metabolism. Furthermore, its accuracy, efficiency, and cost effectiveness make it a promising candidate for broader applications in evaluating the overall health and fitness of the general population. It is crucial to expand its application to target these populations effectively.

Of note, FTIR spectroscopy is remarkably versatile. Thus, among the 15 studies focused on exercise, each had a distinct emphasis, whether it was metabolism, cardiovascular response, muscular fatigue, performance, or general health. Instead of focusing on a specific aspect, it comprehensively analyzes the entire profile, considering the complexity of the human body’s metabolites and proteins. This holistic approach has the potential to lead to new discoveries within the scientific community. Our analysis shows that the use of mathematical models and machine-learning algorithms, particularly a principal component analysis, PLS-DA, and clustering, is closely tied to FTIR analysis success. All these described applications are even more evident from some very recent publications, all of them analyzing the acute response to exercise. Chrimatopoulos et al. have demonstrated the potential of using ATR-FTIR combined with PLS-DA to distinguish between walking, jogging, and running. In addition to highlighting the sensitivity of the method, they were able to identify key salivary metabolites that serve as principal indicators for this classification, including thiocyanate, phospholipids, lactate, phosphate, and glucose [34]. Similarly, Bejar-Grimalt et al. and de Souza et al. employed the same technique to analyze biochemical changes during a 10-km run and to distinguish between continuous exercise, high-intensity interval training, and resistance exercise, respectively [35, 36]. Once again, the studies emphasized the importance of specific wave numbers in differentiating between each type of exercise.

Moreover, there was data evidence of the great potential of FTIR to help to stratify individuals in sport, particularly in regard to classifying high-level athletes. The significance of data analysis methodology is evident and combining it with machine-learning techniques could unlock even greater potential.

Looking to the future and considering ongoing developments, it is essential to explore whether FTIR can be applied across various exercise modalities and different demographic groups, including sedentary individuals. Thus far, its use has been limited to characterizing a sample’s status, and it has yet to be fully harnessed for prescribing exercise plans, encompassing aspects such as frequency, intensity, type, and duration. This endeavor involves evaluating and measuring a multitude of diverse parameters to establish models capable of tracking larger, more intricate exercise cohorts.

## References

[1] Santos AC, Willumsen J, Meheus F, Ilbawi A, Bull FC. The cost of inaction on physical inactivity to public health-care systems: a population-attributable fraction analysis. Lancet Glob Health. 2023;11(1):e32–9. 36480931 10.1016/S2214-109X(22)00464-8 PMC9748301

[2] Petibois C, Cazorla G, Deleris G. FT-IR spectroscopy utilization to sportsmen fatigability evaluation and control. Med Sci Sports Exerc. 2000;32(10):1803–8. 11039657 10.1097/00005768-200010000-00023

[3] Petibois C, Cazorla G, Poortmans JR, Déléris G. Biochemical aspects of overtraining in endurance sports. Sports Med. 2002;32:867–78. 12392446 10.2165/00007256-200232130-00005

[4] Rankovic G, Mutavdzic V, Toskic D, Preljevic A, Kocic M, Nedin Rankovic G, et al. Aerobic capacity as an indicator in different kinds of sports. Bosn J Basic Med Sci. 2010;10(1):44–8. 20192930 10.17305/bjbms.2010.2734 PMC5596610

[5] Haris B. FTIR spectroscopy for analysis of protein secondary structure. In: Haris P, editor. Biological and biomedical infrared spectroscopy. Amsterdam: IOS Press BV; 2009. p. 129–67.

[6] Pupeza I, Huber M, Trubetskov M, Schweinberger W, Hussain SA, Hofer C, et al. Field-resolved infrared spectroscopy of biological systems. Nature. 2020;577(7788):52–9. 31894146 10.1038/s41586-019-1850-7

[7] Haris B. Infrared spectroscopy: past and present. In: Haris P, editor. Biological and biomedical infrared spectroscopy. Amsterdam: IOS Press BV; 2009. p. 1–52.

[8] Tiernan H, Byrne B, Kazarian SG. ATR-FTIR spectroscopy and spectroscopic imaging for the analysis of biopharmaceuticals. Spectrochim Acta A Mol Biomol Spectrosc. 2020;241: 118636. 32610215 10.1016/j.saa.2020.118636 PMC7308041

[9] Huber M, Kepesidis KV, Voronina L, Fleischmann F, Fill E, Hermann J, et al. Infrared molecular fingerprinting of blood-based liquid biopsies for the detection of cancer. Elife. 2021;10: e68758. 34696827 10.7554/eLife.68758 PMC8547961

[10] Page MJ, McKenzie JE, Bossuyt PM, Boutron I, Hoffmann TC, Mulrow CD, et al. The PRISMA 2020 statement: an updated guideline for reporting systematic reviews. BMJ. 2021;372: n71. 33782057 10.1136/bmj.n71 PMC8005924

[11] National Heart LaBI. Quality assessment of systematic reviews and meta-analyses. 2021. Available from: https://www.nhlbi.nih.gov/health-topics/study-quality-assessment-tools. [Accessed Mar 2023].

[12] Petibois C, Déléris G, Cazorla G. Nouvelles perspectives pour le suivi biologique des sportifs 2: prévention du surentraînement par spectrométrie IR-TF. Sci Sports. 2000;15(2):98–100.

[13] Petibois C, Déléris G, Cazorla G. Nouvelles perspectives pour le suivi biologique des sportifs 1: l’analyse métabolique par spectrométrie IR-TF. Sci Sports. 2000;15(2):95–7.

[14] Petibois C, Cazorla G, Cassaigne A, Déléris G. Application of FT-IR spectrometry to determine the global metabolic adaptations to physical conditioning in sportsmen. Appl Spectrosc. 2002;56(10):1259–67.

[15] Petibois C, Paiva M, Cazorla G, Déléris G. Discriminant serum biochemical parameters in top class marathon performances. Jpn J Physiol. 2002;52(2):181–90. 12139776 10.2170/jjphysiol.52.181

[16] Petibois C, Cazorla G, Déléris G. The biological and metabolic adaptations to 12 months training in elite rowers. Int J Sports Med. 2003;24(1):36–42. 12582950 10.1055/s-2003-37194

[17] Petibois C, Déléris G. Fourier-transform infrared spectrometry determination of the metabolic changes during a maximal 400-meter swimming test. Int J Sports Med. 2003;24(5):313–9. 12868040 10.1055/s-2003-40707

[18] Petibois C, Déléris G. Erythrocyte adaptation to oxidative stress in endurance training. Arch Med Res. 2005;36(5):524–31. 16099333 10.1016/j.arcmed.2005.03.047

[19] Petibois C, Déléris G. Evidence that erythrocytes are highly susceptible to exercise oxidative stress: FT-IR spectrometric studies at the molecular level. Cell Biol Int. 2005;29(8):709–16. 15953739 10.1016/j.cellbi.2005.04.007

[20] Petibois C, Déléris G. FT-IR spectrometry analysis of plasma fatty acyl moieties selective mobilization during endurance exercise. Biopolymers. 2005;77(6):345–53. 15739181 10.1002/bip.20196

[21] Khaustova S, Shkurnikov M, Tonevitsky E, Artyushenko V, Tonevitsky A. Noninvasive biochemical monitoring of physiological stress by Fourier transform infrared saliva spectroscopy. Analyst. 2010;135(12):3183–92. 20953513 10.1039/c0an00529k

[22] Vieira C, Pupin B, Bhattacharjee TT, Sakane KK. Infrared spectroscopy based study of biochemical changes in saliva during maximal progressive test in athletes. Anal Sci. 2021;37(8):1157–63. 33518584 10.2116/analsci.20P395

[23] Chrimatopoulos C, Pavlou E, Kourkoumelis N, Sakkas V. Discriminating the salivary profile of athletes using ATR-FTIR spectroscopy and chemometrics. Chemometr Intell Lab Syst. 2022;230: 104660.

[24] Lemes LC, Caetano Júnior PC, Strixino JF, Aguiar J, Raniero L. Analysis of serum cortisol levels by Fourier transform infrared spectroscopy for diagnosis of stress in athletes. Rev Bras Eng Biomed. 2016;32(3):293–300.

[25] Caetano Júnior PC, Aguiar JC, Ferreira-Strixino J, Raniero LJ. Isokinetic muscle performance and salivary immune-endocrine responses in handball players by Fourier transform infrared spectroscopy. Rev Andal Med Deporte. 2017;10(3):125–31.

[26] Caetano PC, Strixino JF, Raniero L. Analysis of saliva by Fourier transform infrared spectroscopy for diagnosis of physiological stress in athletes. Rev Bras Eng Biomed. 2015;31(2):116–24.

[27] Khaustova SA, Shkurnikov MU, Grebenyuk ES, Artyushenko VG, Tonevitsky AG. Assessment of biochemical characteristics of the saliva using Fourier transform mid-infrared spectroscopy. Bull Exp Biol Med. 2009;148(5):841–4. 20396807 10.1007/s10517-010-0831-5

[28] McCord JM, Fridovich I. Superoxide dismutase: an enzymic function for erythrocuprein (hemocuprein). J Biol Chem. 1969;244(22):6049–55. 5389100

[29] Helm D, Labischinski H, Schallehn G, Naumann D. Classification and identification of bacteria by Fourier-transform infrared spectroscopy. J Gen Microbiol. 1991;197:69–79. 10.1099/00221287-137-1-69 1710644

[30] Petibois C, Déléris G. Analysis and monitoring of oxidative stress in exercise and training by FTIR spectrometry. Int J Sports Physiol Perform. 2008;3(2):119–30. 19208921 10.1123/ijspp.3.2.119

[31] Gajjar K, Trevisan J, Owens G, Keating PJ, Wood NJ, Stringfellow HF, et al. Fourier-transform infrared spectroscopy coupled with a classification machine for the analysis of blood plasma or serum: a novel diagnostic approach for ovarian cancer. Analyst. 2013;138:3917–26. 23325355 10.1039/c3an36654e

[32] Wei X, Yu K, Wu D, Huang P, Sun Q, Wang Z. Species identification of semen stains by ATR-FTIR spectroscopy. Int J Legal Med. 2021;2:73–80. 10.1007/s00414-020-02367-0 32647962

[33] Caixeta DC, Lima C, Xu Y, Guevara-Vega M, Espindola FS, Goodacre R, et al. Monitoring glucose levels in urine using FTIR spectroscopy combined with univariate and multivariate statistical methods. Spectrochim Acta A Mol Biomol Spectrosc. 2023;2022(290): 122259. 10.1016/j.saa.2022.122259 36584643

[34] Chrimatopoulos C, Chrimatopoulos G, Sakkas V. Investigating oral biofluid markers as a tool for the chemometric discrimination of different physical exercise intensities utilizing ATR-FTIR spectroscopy. Chemometr Intell Lab Syst. 2023;242: 104990.

[35] Bejar-Grimalt J, Sanchez-Illana A, Guardia M, Garrigues S, Catala-Vilaplana I, Bermejo-Ruiz JL, et al. Dryfilm-ATR-FTIR analysis of urinary profiles as a point-of-care tool to evaluate aerobic exercise. Anal Methods. 2024;16(35):5982–9. 39162061 10.1039/d4ay00913d

[36] de Souza AV, Teixeira RR, Caixeta DC, Silva ATF, Gonçalves LCO, Giolo JS, et al. Salivary spectral signature using ATR-FTIR spectroscopy in different exercise protocols. Spectrochim Acta A Mol Biomol Spectrosc. 2024;320: 124599. 38865886 10.1016/j.saa.2024.124599

[37] Caetano Júnior PC, Lemes LC, Aguiar JC, Strixino JF, Raniero L. Application of FT-IR spectroscopy to assess physiological stress in rugby players during fatigue test. Rev Bras Eng Biomed. 2016;32(2):123–8.

